# Effects of Two Foot-Ankle Interventions on Foot Structure, Function, and Balance Ability in Obese People with Pes Planus

**DOI:** 10.3390/healthcare9060667

**Published:** 2021-06-03

**Authors:** Du-Jin Park, Kyung-Sun Lee, Se-Yeon Park

**Affiliations:** 1 Department of Physical Therapy, College of Health Sciences, Catholic University of Pusan, Busan 46252, Korea; djpark35@cup.ac.kr; 2 Department of Industrial Health, College of Health Sciences, Catholic University of Pusan, Busan 46252, Korea; ksunlee@cup.ac.kr; 3 Department of Physical Therapy, Uiduk University, Gyeongju 38004, Korea

**Keywords:** foot-ankle exercises, obesity, pes planus, short foot, proprioceptive neuromuscular facilitation

## Abstract

Obese people are prone to foot deformities such as flat feet. Foot management programs are important to prevent them. This study investigated the effects of two foot-ankle interventions on balance ability, foot arch, ankle strength, plantar fascia thickness, and foot functions in obese people with pes planus for four weeks. The experiment was designed as a randomized controlled trial. Twenty-four participants who met the inclusion criteria were selected, and they were randomly assigned to either a short foot group (SFG) or proprioceptive neuromuscular facilitation group (PNFG) according to foot-ankle intervention. Two interventions were commenced three times a week for 20 min over four weeks. The tests were conducted at two intervals: pre-intervention and at four weeks. The tests were conducted in the following order: the patient-specific functional scale test (PSFS), an ultrasound of the plantar fascia, the navicular drop test, balance test, and the four-way ankle strength test. Two groups showed significant differences in balance ability, foot arch, ankle strength, plantar fascia thickness, and foot functions between pre-test and post-test (*p* < 0.05). PNFG had significantly higher dorsiflexor and invertor strength than SFG (*p* < 0.05). SF and PNF interventions were effective to improve balance ability, foot arch, ankle strength, plantar fascia thickness, and foot functions in obese people with pes planus. Additionally, PNF intervention is more beneficial in increasing the dorsiflexor and invertor strength compared to SF intervention.

## 1. Introduction

Obesity is a global health problem with a gradually increasing prevalence [1]. In South Korea, the number of obese adults aged >20 years increased from 28.7% in 2006 to 32.4% in 2015 [2]. Body mass index (BMI) is a commonly used parameter for evaluating obesity and is calculated by dividing weight in kilograms by the square of height in square meters [3]. According to the standards of the World Health Organization, a BMI of 18.5–24.9 kg/m^2^ is classified as normal, ≤18.5 kg/m^2^ is classified as underweight, 25.0–29.9 kg/m^2^ is classified as overweight, and ≥30 kg/m^2^ is classified as obesity [4]. As of 2014, the number of overweight individuals aged >18 years was estimated to be >1.9 billion, accounting for 39% of the total global population, and the number of obese individuals was estimated to be >600 million, accounting for 13% of the total global population [5].

Obesity is considered to be the cause of various musculoskeletal problems in both adults and children [6,7]. Particularly, among the musculoskeletal problems, the pes planus deformity is common in obese populations, and increased BMI has been reported to be associated with foot pain and pes planus deformity in children, adolescents, and adults [8,9]. Chougala et al. reported that, according to Dennison’s evaluation method, 44% of young adults are predisposed to the risk of pes planus [10]. Moreover, in the case of young adults, there is a high correlation between BMI and change in the height of the foot arch [11], and a previous study reported that >78% of adults with pes planus are overweight [12]. In addition, a recent study in young adults reported that obese individuals are susceptible to developing plantar fasciitis owing to increased thickness of the plantar fascia and decreased height of the foot arch compared with their normal weight counterparts [13]. As described above, even in the case of young adults, obesity can cause changes in the structure and function of the feet. Thus, preventive interventions are needed.

Short-foot (SF) exercise, which is a basic exercise method that can activate the internal muscles called the foot core, is a representative intervention for pes planus [14]. In previous studies, SF exercise for six weeks improved the height of the foot arch, pain in the foot, and function of the foot in patients with pes planus [15], and SF exercise for four weeks also resulted in a decrease in the height difference of the foot arch during the navicular drop test [16]. Recently, an intervention method based on proprioceptive neuromuscular facilitation (PNF) leg patterns has been introduced as a method for harmoniously strengthening the activities of not only the intrinsic muscles but also the external muscles of the foot [17,18,19]. Recent electromyographic studies have proven that a three-dimensional foot-ankle exercise using the PNF leg patterns is as effective as the SF and toe-spread-out exercises in training the intrinsic muscles [19]. In addition, the application of a three-dimensional foot-ankle exercise for four weeks in obese people with plantar fasciitis resulted in improvements of foot function, pain, and ankle strength [18]. Obese people have weaker ankle evertor muscles than normal-weight people, and weakness in this muscle group increases the risk of ankle sprains [13]. To increase the stability of the foot, coordinated activity of the internal and external muscles is essential [14], and ankle strength is a predictive factor for the stability and balance ability of the foot [20].

Furthermore, recent findings have identified the clinical value of PNF in several ankle diseases [18,21]. A three-dimensional foot-ankle intervention using the PNF leg patterns, which can strengthen both the internal and external reinforcement of the foot with respect to SF exercise, is warranted. Therefore, the present study aimed to analyze and compare the effects of two foot-ankle interventions on balance ability, foot arch, ankle strength, plantar fascia thickness, and foot function in obese people with pes planus for four weeks.

## 2. Methods

### 2.1. Participants

To achieve an appropriate sample size, a pilot study was conducted consisting of three participants in each of the two groups. Changes in evertor strength were measured and compared between a short foot group (SFG) (0.20 ± 0.15) and proprioceptive neuromuscular facilitation group (PNFG) (0.46 ± 0.38). As a result, the effect size was 0.90, which was used to perform a G-Power analysis. The number of participants in this study was set according to 0.9 effect size, 0.8 power, and significance level of 0.05, following the experimental design, and was calculated using G-Power analysis (University of Dusseldorf, Dusseldorf, Germany). The recruitment announcement for this study was posted on the bulletin boards inside and outside the university. Study participants were collected through a recruitment announcement, and a total of 44 participants were recruited. This study was conducted in the Kaya University laboratory in Gimhae. Among the recruited young men and women, only those who satisfied the inclusion criteria and who fully understood and agreed to the research processes and purpose were selected. The selection was limited to those with a BMI of ≥25 kg/m^2^ [22], those with a height difference of ≥ 10 mm during the navicular drop test [23], and those with an inner longitudinal arch angle of ≥ 150° [24]. In addition, those with other foot deformities or diseases, inability to perform exercise, pain around the feet and ankles, and neurological disorders were excluded from the study. Twenty-four participants who met the inclusion criteria were selected for the study. The entire study procedure was approved by the Kaya University Institutional Review Board (Kaya IRB-203) and complied with the Helsinki Declaration.

### 2.2. Study Design

**Figure 1:**
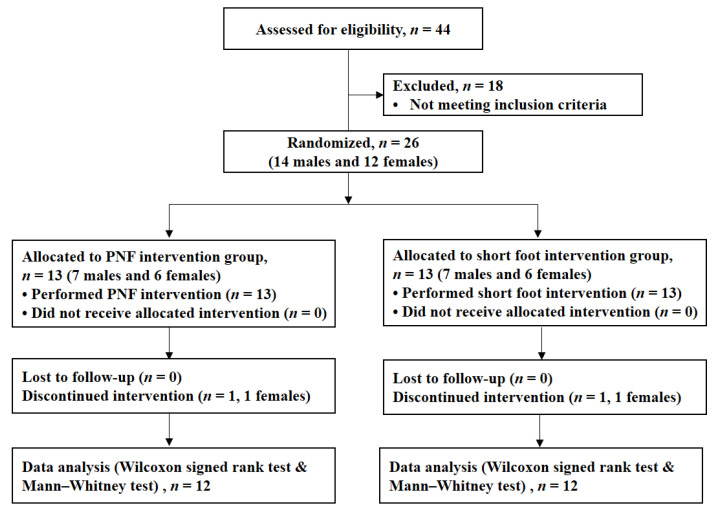
Flow diagram of participants in this study. PNF, proprioceptive neuromuscular facilitation.

This study was a single-blinded, randomized controlled trial. Blocked randomization was performed based on numbers generated using Excel 2016 software (Microsoft Office Professional Plus 2016, Microsoft Corp., Redmond, WA, USA). The code for each group was sealed in opaque envelopes, and an independent researcher assigned the participants to either a short foot group (SFG) or a proprioceptive neuromuscular facilitation group (PNFG). The participants were blinded by a researcher-unrelated measurement and intervention for group assignment. However, an examiner was involved in both the intervention supervision and measurements of the dependent variables. Intervention training for the participants was performed by the same physical therapist, who had more than 10 years of experience. Measurement orders were randomized using Excel software. Flow diagram of this study is presented in Figure 1.

### 2.3. Measurement Methods and Tools

#### 2.3.1. Four-Way Ankle Strength Test

Dorsiflexion, plantar flexion, inversion, and eversion were performed to measure the muscle strength of the ankle joint. Muscle strength was evaluated according to the literature [25]. The participants were positioned in the supine position with the dominant foot over the edge of the table and the ankle in a neutral position. Dorsiflexor and evertor muscles were measured in the direction of the top of the foot, and the plantar flexor and invertor muscles were measured in the direction of the sole. Dorsiflexors were measured by applying resistance to the medial side of the dorsal foot. Evertor strength was tested for resistance to the lateral edge on the dorsal side of the foot. The plantar flexor was achieved by applying resistance to the head of the metatarsal bone on the plantar side of the foot. The invertor was tested with resistance to the lateral edge of the plantar side of the foot. The muscle strength test was performed using a portable competency meter (Commander Muscle Tester; J-Tech Medical Inc., Midvale, UT, USA). To standardize the ankle muscle strength, the muscle strength (N) displayed on the portable competency meter was normalized to the participant’s weight (kg) [13,18].

#### 2.3.2. Navicular Drop Test

To measure the height of the inner longitudinal arch, a navicular drop test was performed. The distance between the participant’s navicular tuberosity and the ground was measured in the sitting and standing positions. Pes planus was diagnosed when the difference in the height of the navicular bone between the sitting position (non-weight support position) and the standing position (weight support position) was >10 mm [23].

#### 2.3.3. Balance Test

A Tekscan pressure mapping tool (Tekscan Inc., South Boston, MA, USA) was used to measure the change in the center of pressure (COP) during one-foot stand-up. The one-foot stand-up movement was performed for 5 s, and the changes in pressure and COP for 3 s, excluding the first and last seconds, were used in the analysis. All measurements were performed for a total of three times each.

#### 2.3.4. Plantar Fascia Thickness Test

To measure the change in the thickness of the plantar fascia before and after the intervention, an ultrasound imaging device (ProSound 2; Hitachi Aloka Medical, Tokyo, Japan) was used. The thickness of the plantar fascia was measured by placing a 6–13 MHz straight transducer vertically on the inner side of the heel bone nodule while the participants were in a prone position with their knees stretched and the dominant foot exposed to the edge of the treatment table, placing the ankle joint in a neutral position [26]. Ultrasonic images and captures were measured in B-mode and static condition.

#### 2.3.5. Questionnaire for Foot Function

**Figure 2:**
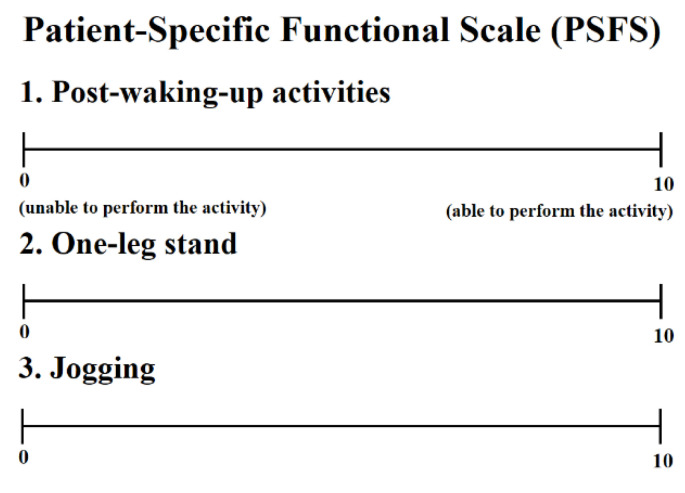
Questionnaire for foot function.

The patient-specific functional scale (PSFS) is a tool designed for easy use by any individual without sacrificing the validity and reliability in evaluating the musculoskeletal function of various patients in clinical practice [27]. Three to five major activities that the patient cannot perform or has difficulty in performing are evaluated. A score of 0 points is assigned if the activity is impossible to perform, and a score of 10 points is assigned if the activity is possible to perform at the pre-injury level [28]. In this study, the level of foot function was measured during three major activities through interviews with participants [18,29]. Among the three main activities, the first item (PSFS 1) was post-waking-up activities, the second item (PSFS 2) was one-leg stand, and the third item (PSFS 3) was jogging (Figure 2).

### 2.4. Experimental Procedure

**Figure 3:**
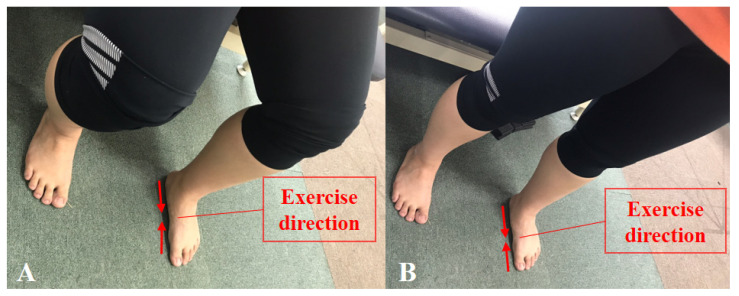
Short foot exercise: (**A**) sitting position; (**B**) standing position.

Before the intervention, all participants performed foot and ankle stretching for 5 min to relieve muscle tone and prevent spasms. Training on the intervention was provided for about 20 min, and only those who passed the training performed the experiment. Each participant carried out the exercise under supervision. The SF exercise was accomplished in two stages (Figure 3). An exercise was performed first to shorten the foot in the forward and backward directions along with an attempt to move the metatarsal heads toward the heel without bending the toes. Thereafter, the previous step was performed again by applying equal load to the three support points of the foot [30]. After performing the SF exercise for 15 s, a rest interval of 15 s was provided, and one set consisted of four repetitions. The rest time between sets was 1 min, and the SF exercise was performed for about 15 min for a total of five sets. Interventions were performed in the sitting position for weeks 1 to 2 and in the standing position for weeks 3 to 4.

**Figure 4:**
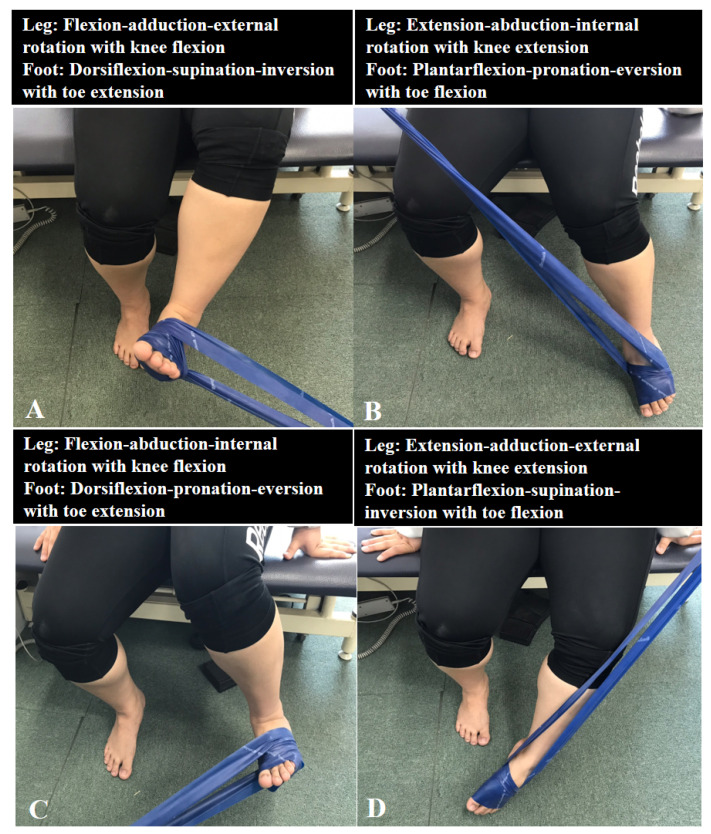
PNF exercise: (**A**) diagonal 1 flexion, (**B**) diagonal 1 extension, (**C**) diagonal 2 flexion, and (**D**) diagonal 2 extension.

For PNF, the bending and extension patterns of the diagonal 1 (D1) and diagonal 2 (D2) leg patterns were alternately performed [17]. The starting position of D1 flexion was leg extension-abduction-internal rotation, together with foot plantar flexion-pronation-eversion with toe flexion. Leg flexion-adduction-external rotation and foot dorsiflexion-supination-inversion with toe extension were performed (Figure 4A). The PNF intervention started with the movement of the toe, which is the distal part. The D1 extension movement was the opposite of the D1 bending movement (Figure 4B). The starting position of D2 flexion was leg plantar flexion-supination-inversion with toe flexion, together with extension-adduction-external rotation. Leg flexion-abduction-internal rotation with knee flexion and foot dorsiflexion-pronation-eversion with toe extension were performed (Figure 4C). The D2 extension exercise was performed the opposite way (Figure 4D). All PNF interventions were performed using an elastic band, and the strength of the resistance of the elastic band was selected according to the 12-repetition maximum. Twelve repetitions were set as one set, 1 min per set, and the rest time after each set was fixed to 30 s. Two sets of each pattern, for a total of eight sets, were performed. After the end of the two sets of each pattern, an additional rest interval of 1 min was provided. The PNF intervention was also performed in the sitting position for weeks 1 to 2 and in the standing position for weeks 3 to 4.

### 2.5. Data Analysis

In this study, the Mann–Whitney test was performed to determine the differences in general characteristics between the two groups. To compare the results before and after the intervention, the Wilcoxon signed rank test was performed. In addition, the Mann–Whitney test was used to compare the changes before and after the intervention between groups. The effect sizes (*r*-value) of the comparisons with statistically significant differences were also calculated as follows: *r* = Z/√N where z is the z-score. For statistical analysis, SPSS 25.0 for Windows (SPSS Inc., Chicago, IL, USA) was used, and the statistical significance level was set to 0.05.

## 3. Results

### 3.1. General Characteristics of All Participants

The general characteristics of all participants are shown in Table 1. There were no significant differences in general characteristics between the two groups (*p* > 0.05).

### 3.2. Comparison of Results before and after Intervention

The SF intervention significantly improved the anterior-posterior and left-right balance, height difference in the navicular drop test, plantar flexor and invertor muscle strength, plantar fascia thickness, and foot function compared with before the intervention (*p* < 0.05) (Table 2). There were no significant differences between groups in dorsiflexor and evertor strength before and after SF intervention (*p* > 0.05) (Table 2). In the PNF intervention, the left-right balance, height difference in the navicular drop test, four-way ankle strength, plantar fascia thickness, and foot function were significantly improved after the intervention compared with before the intervention (*p* < 0.05) (Table 3). There was no significant difference between groups in anterior-posterior balance before and after PNF intervention (*p* > 0.05) (Table 3). The PSFS-itemized scores before and after interventions were as follows: In the SFG, the PSFS 1 scores were 8.00 ± 1.41, 9.17 ± 1.03, and 8.83 ± 1.27 points before intervention, and the PSFS 1-3 scores were 8.75 ± 1.14, 9.83 ± 0.58, and 9.50 ± 0.90 points after intervention, respectively. In the PNFG, the PSFS 1–3 scores were 8.08 ± 1.88, 9.08 ± 1.16, and 9.08 ± 1.16 points before intervention and 9.33 ± 1.23, 9.67 ± 0.78, and 9.83 ± 0.58 points after intervention, respectively.

### 3.3. Comparison of Changes before and after Interventions

**Figure 5:**
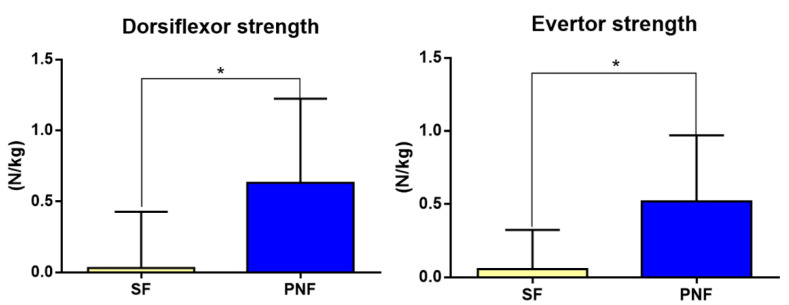
Comparisons of dorsiflexor strength and evertor strength between the short-foot (SF) and proprioceptive neuromuscular facilitation (PNF) interventions. * *p* < 0.05.

Table 4 shows the differences in the changes between before and after the SF and PNF interventions. There were no significant differences in anterior-posterior and left-right balance, height difference in the navicular drop test, plantar flexor and invertor muscle strength, plantar fascia thickness, and foot function between the two groups (*p* > 0.05). The PNF intervention significantly increased the strength of the evertor and dorsiflexor muscles compared with the SF intervention (Z = −2.31, *p* < 0.05, Z = −2.54, *p* < 0.01) (Figure 5).

### 3.4. Intra and Inter-Rater Reliability of Measurements of Plantar Fascia

This study used intra-class correlation coefficients (ICC) to investigate the intra-rater reliability of the measurement of the thickness of the plantar fascia. Ultrasound measurements of plantar fascia thickness showed inter- and intra-rater reliability of 0.89 (0.70–0.98 95% confidence interval) and 0.93 (0.78–0.98 95% confidence interval), respectively (Table 5).

## 4. Discussion

A previous study reported that the application of the SF intervention for four weeks significantly improved the back-and-forth and left-right sway in healthy participants [31]. When the SF intervention was applied for eight weeks in healthy adults, the dynamic balance ability was also improved [32]. In this study, the back-and-forth and left-right sway of obese participants were significantly improved after, compared with before, the SF intervention, supporting the results of the previous study. The SF exercise has been recommended as an initial training to recover the proprioceptive sensation after an ankle injury [33] and is effective in improving the positional and vibrational sensations of ankle eversion [32]. In addition, the intrinsic muscles of the foot activate the muscle spindle in a standing position when a load is applied, and training of the intrinsic muscles in this position can further stimulate the proprioceptive sensation [34]. In this study, the SF intervention was applied in the standing position from week three. The intervention improved the balance ability, possibly by increasing the stimulation of proprioceptive sensations, including positional sensations.

Obese individuals have greater anterior-posterior sway than normal-weight individuals [13]. No significant difference was observed in the change in anterior-posterior balance ability between the SF and PNF groups. However, no significant difference was observed in the anterior-posterior sway between before and after the PNF intervention. Similar results were obtained in the second item of the PSFS. PSFS 2 investigated the difficulty of performing one-foot standing. Both groups had no major problems. The SF intervention resulted in an increase of about 7.2%, from 9.17 to 9.83 points, after four weeks. Meanwhile, the PNF intervention resulted in an increase of about 6.5%, from 9.08 points to 9.67 points. Both groups did not show great difficulty in standing on one foot. However, considering the results of previous studies [31,32] and the changes in balance ability and difficulty when standing on one foot, the SF intervention may be a better alternative to restore balance ability in obese participants. Obesity and foot pronation can increase the risk of chronic heel pain [35], and one of the risk factors for running-related injuries is a pronated foot [36]. A pronated foot posture cannot make the foot rigid compared with the neutral posture, which results in the reduction of the torque of the most concentric plantar flexors [37]. For this condition, the SF intervention is mainly applied. Mulligan and Cook reported an increase in the height of the foot arch and a decrease in the pronated range after applying the SF intervention for four weeks [38]. A recent study recommended applying the SF intervention for four weeks to improve excessive foot pronation by reducing the height difference in the navicular drop test [16,39]. The SF intervention also reduced the height difference in the navicular drop test in our study, causing an increase in the height of the foot arch during weight bearing.

The PNF intervention was as effective as the SF intervention in improving the height of the foot arch and even reduced the height difference in the navicular drop test more than did the SF intervention. This may be closely related to the result that the PNF intervention improved the strength of the dorsiflexor and evertor muscles more than did the SF intervention. The effect sizes of dorsiflexor (*r* = −0.67) and evertor (*r* = −0.73) were also 0.5 or higher. The *r*-value means of 0.1 indicate low effects, 0.3 indicate medium effects, and 0.5 and higher indicate large effects [40]. This result showed that PNF is more benefit for muscle strength of two muscles than SF. The intrinsic muscles of the foot are related to shock absorption, maintenance of the foot arch, and generation of force and torque during gait [41]. However, for the stability of the foot, a coordinated activity of the intrinsic and extrinsic muscles is essential [14]. Particularly, obese individuals show weakness of the evertor muscles of the foot, which is an intrinsic muscle, compared with their normal-weight counterparts [13]. In those with pes planus, the activity of the peroneus longus, an extrinsic muscle, is decreased during gait and the SF exercise [42,43]. The functions of the peroneus longus are plantar flexion and eversion [44]. During the PNF intervention, the D1 extension pattern can activate the peroneus longus through plantar flexion and eversion [17].

In addition, the flexor hallucis brevis, which is an intrinsic muscle that is considered important in maintaining the inner longitudinal arch, can be activated through big toe flexion [45], and toe flexion is a traditional method for measuring the strength of the intrinsic muscles [46]. In addition, the cross-sectional area of the flexor hallucis brevis is decreased in individuals with pes planus compared with their normal counterparts [47]. During the PNF intervention, the extension pattern is able to activate these muscles by bending all toes, including the big toe. As the enhancement of toe flexor muscle strength improves functional movements, such as vertical jumping and running [30,48], the PNF intervention can be applied not only to obese individuals in the general population but also to athletes.

Previous case studies of plantar fasciitis reported a mean fascia thickness of 4.8 mm [49]. In this study, although both groups had no heel pain, the mean thickness of the plan-tar fascia was more than 4.8 mm. Based on the result of a previous study, obese people with pes planus have the potential for developing plantar fasciitis. Weight loss and intervention programs were recommended for them [13]. Both interventions significantly improved the plantar fascia thickness before and after intervention, which shows that both interventions may be helpful for the management of plantar fasciitis cases.

Obese individuals are susceptible to plantar fasciitis development and ankle sprains. In particular, the trigger points of the gastrocnemius are involved in heel pain, which can be a cause of plantar fasciitis [50]. In a previous study, after applying the ankle pattern and isotonic combination of the PNF intervention in obese participants with plantar fasciitis, the foot function, heel pain, and muscle strength of the dorsiflexor and evertor muscles were improved by strengthening the elongation and intrinsic muscles of the plantar fascia [18]. These results support the previous study [51] that reported that stretching of the calf muscle and plantar fascia has moderate effects on plantar fasciitis.

Among the muscles responsible for ankle strength, the evertor muscle is crucial in preventing ankle sprain [52]. During the PNF intervention in this study, the D2 bending pattern not only strengthened the corresponding muscle strength through dorsiflexion and eversion but also elongated the calf muscles and plantar fascia. Through this mechanism, the PNF intervention may improve the support of the inner longitudinal arch by reducing the tension of the plantar fascia and increasing the strength of both muscles compared with the SF intervention. Therefore, the PNF intervention was considered to have resulted in a further reduction in the height difference in the navicular drop test compared with the SF intervention. The third item of the PSFS is related to the difficulty of performing the jogging movement. When examining the results of PSFS 3, the PNF intervention increased the score by 8.26% from 9.08 points before intervention to 9.83 points after intervention, and the SF intervention increased the score by 7.59% from 8.83 to 9.50 points. This result shows that both the PNF and SF interventions can have a positive effect on dynamic activities, such as sports, by strengthening the external muscles, including the dorsiflexor and evertor muscles. On the basis of the results of this study, the PNF intervention is a preventive strategy that can reduce the risk for various foot-related diseases in obese individuals with pes planus.

Some limitations of the study need to be acknowledged. Focusing on changes in the foot, BMI change was not measured after the intervention. Based on the characteristics of Asians, the incidence of obesity among young people is lower in Korea than that in the United States or Europe. There are many restrictions on recruiting obese young adults in Korea, one of which is their shameful perception of being obese. Therefore, the sample size for this study was small, making it difficult to generalize the findings. Dynamic physical activity is restricted by foot deformities or dysfunctions in obese people. However, changes in dynamic physical activity were not observed after a four-week intervention.

## 5. Conclusions

Both the SF and PNF interventions, when applied for four weeks, are effective in restoring balance, foot arch height, ankle muscle strength, plantar fascia thickness, and foot function in obese individuals with pes planus. The PNF intervention is more effective than the SF intervention in strengthening the dorsiflexor and evertor muscles in obese persons with pes planus.

## Figures and Tables

**Table 1:** Descriptive statistics of participants (*n* = 24).

Variable	SF (*n* = 12)	PNF (*n* = 12)	Z	*p*
Age (years)	23.25 ± 1.22	24.00 ± 1.48	−1.60	0.11
Height (cm)	168.50 ± 8.44	170.25 ± 10.06	−0.46	0.64
Weight (kg)	83.83 ± 13.83	85.88 ± 19.99	−0.12	0.91
BMI (kg/m^2^)	29.34 ± 2.81	29.39 ± 4.57	−0.40	0.69
PSFS (score)	26.00 ± 3.44	26.25 ± 3.98	−0.36	0.76
Sex	M 7 (50%), F 5 (50%)	M 7 (50%), F 5 (50%)	-	-
Dominant foot	Rt 10 (83.3%), Lt 2 (16.7%)	Rt 10 (83.3%), Lt 2 (16.7%)	-	-

SF, short foot exercise; PNF, proprioceptive neuromuscular facilitation exercise; M, male; F, female; Rt, right; Lt, left; BMI, body mass index; PSFS, patient-specific functional scale.

**Table 2:** Comparison of results between pre-test and post-test in the SF group (*n* = 12).

SF	Pre-Test	Post-Test	Z	*p*	Effect Size (*r*)
COP-LR (cm)	3.20 ± 1.24	2.23 ± 0.72	−2.43	0.02	−0.70
COP-AP (cm)	2.21 ± 0.65	1.63 ± 0.48	−2.98	0.01	−0.86
NDT (mm)	11.83 ± 1.75	9.17 ± 1.64	−2.73	0.01	−0.79
Dorsiflexor (N/kg)	2.29 ± 0.66	2.32 ± 0.65	−0.31	0.75	−0.09
Plantar flexor (N/kg)	2.31 ± 0.42	2.76 ± 0.39	−2.51	0.01	−0.72
Invertor (N/kg)	1.10 ± 0.32	1.28 ± 0.25	−2.59	0.01	−0.75
Evertor (N/kg)	1.34 ± 0.37	1.39 ± 0.26	−0.78	0.43	−0.23
PFT (cm)	0.50 ± 0.07	0.48 ± 0.07	−2.73	0.01	−0.79
PSFS (scores)	26.00 ± 3.44	28.08 ± 1.83	−2.39	0.02	−0.69

SF, short-foot exercise; COP-LR, center of pressure-left and right; COP-AP, center of pressure-anterior and posterior; NDT, navicular drop test; PFT, plantar fascia thickness; PSFS, patient-specific functional scale; N, newton.

**Table 3:** Comparison of results between pre-test and post-test in the PNF group (*n* = 12).

PNF	Pre-Test	Post-Test	Z	*p*	Effect Size (*r*)
COP-LR (cm)	3.25 ± 1.02	2.18 ± 0.86	−2.67	0.01	−0.77
COP-AP (cm)	2.09 ± 0.56	1.66 ± 0.55	−2.08	0.38	−0.60
NDT (mm)	11.92 ± 1.68	8.67 ± 1.78	−2.95	0.01	−0.85
Dorsiflexor (N/kg)	2.37 ± 0.68	3.00 ± 0.78	−2.75	0.01	−0.79
Plantar flexor (N/kg)	2.81 ± 0.70	3.40 ± 0.49	−2.67	0.01	−0.77
Invertor (N/kg)	1.27 ± 0.41	1.51 ± 0.39	−2.35	0.02	−0.68
Evertor (N/kg)	1.49 ± 0.47	2.01 ± 0.56	−2.75	0.01	−0.79
PFT (cm)	0.51 ± 0.06	0.48 ± 0.06	−3.01	0.01	−0.87
PSFS (scores)	26.25 ± 3.98	28.83 ± 2.21	−2.38	0.02	−0.69

PNF, proprioceptive neuromuscular facilitation; COP-LR, center of pressure-lateral; COP-AP, center of pressure-anteroposterior; NDT, navicular drop test; PFT, plantar fascia thickness; PSFS, patient-specific functional scale; N, newton.

**Table 4:** Comparison of results between the SF and PNF groups (*n* = 24).

Difference	SF	PNF	Z	*p*	Effect Size (*r*)
COP-LR (cm)	−0.98 ± 1.28	−1.07 ± 1.06	−0.69	0.51	−0.20
COP-AP (cm)	−0.58 ± 0.40	−0.43 ± 0.57	−0.49	0.63	−0.14
NDT (mm)	−2.67 ± 2.57	−3.25 ± 2.01	−0.88	0.41	−0.25
Dorsiflexor (N/kg)	0.03 ± 0.39	0.63 ± 0.59	−2.31	0.02	−0.67
Plantar flexor (N/kg)	0.45 ± 0.48	0.59 ± 0.61	−0.58	0.59	−0.17
Invertor (N/kg)	0.18 ± 0.20	0.25 ± 0.28	−0.64	0.55	−0.18
Evertor (N/kg)	0.05 ± 0.27	0.52 ± 0.45	−2.54	0.01	−0.73
PFT (cm)	−0.02 ± 0.02	−0.03 ± 0.01	−0.74	0.48	−0.21
PSFS (scores)	2.08 ± 2.31	2.58 ± 2.68	−0.33	0.76	−0.10

SF, short-foot exercise; PNF, proprioceptive neuromuscular facilitation; COP-LR, center of pressure-lateral; COP-AP, center of pressure-anteroposterior; NDT, navicular drop test; PFT, plantar fascia thickness; PSFS, patient-specific functional scale.

**Table 5:** Intra and inter-rater reliability of measurements of plantar fascia (*n* = 8).

Reliability	Inter-Rater		Intra-Rater	
	ICC_2,1_	95% CI	ICC_3,1_	95% CI
Plantar fascia	0.89	0.70–0.98	0.93	0.78–0.98

ICC, intra-class correlation coefficients; CI, confidence interval.

## Data Availability

The data presented in this study are available on request from the corresponding author. The data are not publicly available due to privacy reasons.
